# Development of Self-Assembled Biomimetic Nanoscale Collagen-like Peptide-Based Scaffolds for Tissue Engineering: An In Silico and Laboratory Study

**DOI:** 10.3390/biomimetics8070548

**Published:** 2023-11-14

**Authors:** Beatriz G. Goncalves, Ryan M. Heise, Ipsita A. Banerjee

**Affiliations:** Department of Chemistry, Fordham University, 441 East Fordham Road, Bronx, New York, NY 10458, USA; bgoncalves1@fordham.edu (B.G.G.); rheise@fordham.edu (R.M.H.)

**Keywords:** scaffolds, peptide-based, nanoscale, self-assembly, tissue engineering

## Abstract

Development of biocomposite scaffolds has gained tremendous attention due to their potential for tissue regeneration. However, most scaffolds often contain animal-derived collagen that may elicit an immunological response, necessitating the development of new biomaterials. Herein, we developed a new collagen-like peptide,(Pro-Ala-His)_10_ (PAH)_10_, and explored its ability to be utilized as a functional biomaterial by incorporating it with a newly synthesized peptide-based self-assembled gel. The gel was prepared by conjugating a pectin derivative, galataric acid, with a pro-angiogenic peptide (LHYQDLLQLQY) and further functionalized with a cortistatin-derived peptide, (Phe-Trp-Lys-Thr)_4_ (FWKT)_4_, and the bio-ionic liquid choline acetate. The self-assembly of (PAH)_10_ and its interactions with the galactarate-peptide conjugates were examined using replica exchange molecular dynamics (REMD) simulations. Results revealed the formation of a multi-layered scaffold, with enhanced stability at higher temperatures. We then synthesized the scaffold and examined its physicochemical properties and its ability to integrate with aortic smooth muscle cells. The scaffold was further utilized as a bioink for bioprinting to form three-dimensional cell-scaffold matrices. Furthermore, the formation of actin filaments and elongated cell morphology was observed. These results indicate that the (PAH)_10_ hybrid scaffold provides a suitable environment for cell adhesion, proliferation and growth, making it a potentially valuable biomaterial for tissue engineering.

## 1. Introduction

Collagen is one of the most copious proteins in the human body and is a major component of bone, muscle, tendon, skin and other connective tissue [1]. It plays a vital role as an extracellular matrix protein and provides structural integrity and support for both hard and soft tissues [2]. It is also utilized as a desired biomaterial for the development of scaffolds for tissue engineering [3]. Each polypeptide chain of collagen consists of repeats of the following sequence: Gly-X-O, where X is usually occupied by L-proline (Pro) and O is hydroxy-l-proline (Hyp). Each of the polypeptide chains is wrapped into triple helices, which gives collagen its robustness and stability [4]. It is generally extracted and purified from various animal sources for use in biological applications [5]. Several gelatinous scaffold biomaterials derived from animal-derived collagen have been developed for wound healing, tissue homeostasis and bioengineering of various tissues including bone, cartilage, skin, cardiac tissue and others [6,7,8]. While there are several animal and fish sources utilized to extract collagen, the most common species employed is cattle (bovine) [9]. However, in some cases, collagen-derived grafts developed from those sources have been found to cause immunogenicity or infection [10,11]. Thus, animal-free scaffolds such as plant-derived scaffolds [12], and micro- and nanoscale and synthetic polymers, are being developed [13]. In particular, collagen mimics or other synthetic derivatives of collagen have shown promise due to their ability to mimic natural collagen and self-assemble into fibrous ordered nano- and microstructures that can be fine-tuned for biological applications [14,15].

In their pioneering work, Conticello, Chaikof and co-workers developed a novel collagen mimetic peptide (CMP) sequence (Pro-Arg-Gly)_4_(Pro-Hyp-Gly)_4_(GluHypGly)_4_ by utilizing ion-pair interactions, which promoted self-assembly into fibrous matrices that closely resembled native collagen fibers. They inserted positively charged residues at the N-terminal end and negatively charged residues at the C-terminal end [16]. In a study conducted by Kiick and co-workers, phenylalanine and pentafluorophenalanine were conjugated with (Gly-Pro-Hyp)_10_ at the N- and C-terminals, respectively, to form CMPs that formed fibrous matrices. Furthermore, those CMPs were found to stimulate platelet aggregation, indicating their potential applicability as biomaterials [17]. In another interesting study, Hartgerink and co-workers developed heterotrimeric CMPs where the three peptide fragments consisting of (Pro-Lys-Gly)_10_, (Asp-Hyp-Gly)_10_ and (Pro-Hyp-Gly)_10_ efficiently formed triple helices with high thermal stability due to charged pair interactions between Asp and Lys residues [18]. Hybrid CMPs have also been devised consisting of four-armed polyethylene glycol (PEG)-cross-linked collagen mimetic peptide hydrogels with biomechanical properties comparable to natural collagen and fibrin gels [19]. In a separate study, dendrimers such as polyamidoamine (PAMAM) were decorated with CMPs that demonstrated facile integrin-mediated cell adhesion [20]. In another study, self-assembled pentafluoro-F(GPO)_4_GPK(GPO)_5_F-based CMP scaffolds were prepared that were utilized as templates for the formation of gold nanowires [21]. In previous work, Kasznel and co-workers developed the sequence (POG)_2_-(azaPOG)-(PRazG)-(POG)_4_ (where azaP and azaG indicate aza-proline and aza-glycine, respectively), which could successfully mimic the stereochemistry of the amino acids and folding seen in natural triple helix collagen [22].

In previous work, we demonstrated through molecular dynamics simulations and docking that the short peptide (Pro-Ala-His)_3_ self-assembled and adhered to collagen models [23]. To further explore its applicability for potential tissue engineering applications, we have carried out replica exchange molecular dynamics (REMD) and laboratory studies of a longer version of this collagen-like peptide sequence, specifically (Pro-Ala-His)_10_.

We chose the (PAH)_10_ sequence as histidine contains an imidazole ring that can promote self-assembly through stacking and CH-π interactions while alanine has been shown to have the propensity to promote helical structures, ref. [24], and proline was maintained as a part of the collagen sequence. Furthermore, we utilized a layer-by-layer assembly approach to create a biomimetic scaffold that can potentially mimic the components of the extracellular matrix and promote cell adhesion and proliferation. Specifically, the hydrophilic natural product galactaric acid was conjugated with the angiogenic vascular endothelial growth factor (VEGF) mimetic peptide sequence LHYQDLLQLQY [25], self-assembled and functionalized with the anti-inflammatory peptide (FWKT)_4_. This tetramer sequence is part of a derivative of the neuropeptide cortistatin that has been known to mimic somatostain, which plays several important physiological roles [26,27,28,29].

In a study conducted by Lauritano and co-workers, it was shown that somatostatin upregulated bone morphogenic protein BMP4 and transformed growth factor receptor (TGFβ) genes and stimulated the self-renewal of dental pulp stem cells and thus may have applications in tissue regeneration [30]. In particular, cortistatin has been found to function as an anti-inflammatory peptide [31] and slow down the development and progression of osteoarthritis [32]. Thus, we hypothesized that the incorporation of the (FWKT)_4_ sequence into the biomimetic scaffold would impart anti-inflammatory properties to the biomimetic composite. Following the functionalization with (FWKT)_4_, we incorporated the collagen-like peptide (Pro-Ala-His)_10_ by layer-by-layer assembly to mimic the components of ECM. As a final layer, the biomimetic ionic liquid choline acetate was incorporated to impart electrical and gelatinous properties to the scaffold. Choline acetate was selected as the incorporation of trace amounts of bio-ionic liquids with peptide-based assemblies that have been shown to form gelatinous assemblies [33,34].

**Scheme 1:**
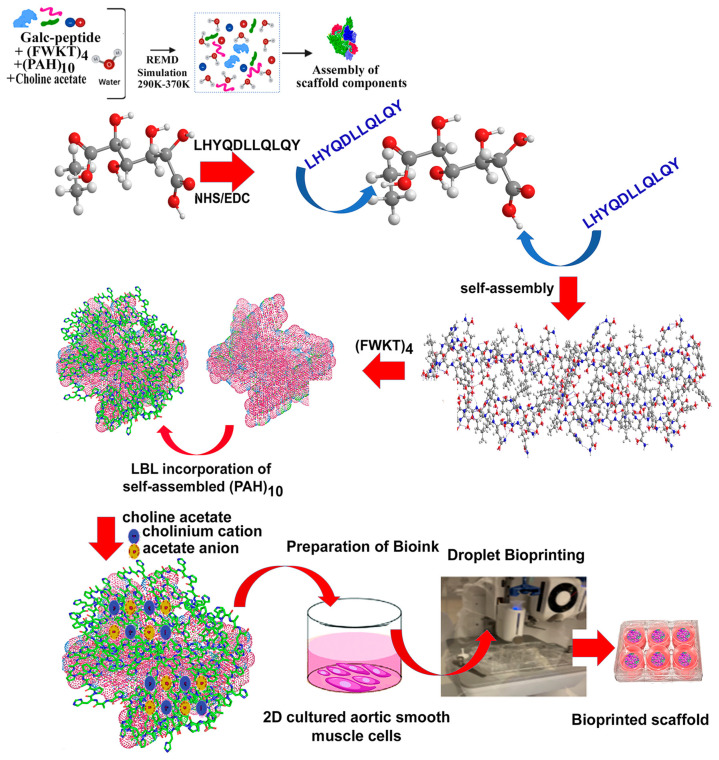
REMD simulation of components utilized in the formation of the scaffold, including collagen-like peptide (PAH)_10_, GalC-peptide conjugate, (FWKT)_4_ and choline acetate followed by synthesis, assembly and bioprinting of aortic smooth muscle cell-laden scaffold.

The scaffold material was then utilized as a bioink component and bioprinted along with aortic smooth cells to develop bioprinted scaffolds that were found to encourage the growth, proliferation and incorporation of the cells into the multilayered matrices. The scheme for the formation of the scaffold is shown in Scheme 1. Thus, we have developed a new self-assembled collagen-like peptide (PAH)_10_, which was functionalized to form a novel scaffold that may be utilized for potential therapeutic applications as an alternative biocomponent for tissue engineering.

## 2. Materials and Methods

### 2.1. Computational Methods

#### 2.1.1. I-Tasser

For protein structure prediction of the collagen-like peptide (PAH)_10_, the web server i-Tasser was utilized. Briefly, i-Tasser is a platform that allows for automated protein structure and function prediction based on an inputted sequence. It refers to databases by PSI-BLAST to identify evolutionary similarities at which point it then outputs an estimate of accuracy for the predictions given [35].

#### 2.1.2. Replica Exchange Molecular Dynamics of (PAH)_10_

We first conducted REMD simulations to study the self-assembly of (PAH)_10_ assemblies. The software DESMOND from Schrödinger was utilized in the Maestro Material Sciences suite (2021-3) platform [36]. The Disordered System Builder application was then applied to build a randomized multi-component system. Five (PAH)_10_ strands along with 6000 water molecules were added into an initial box size of 10 Å × 10 Å × 10 Å. The initial placement of each component was then selected to be amorphous, which builds the cell by placing each component individually at random locations and orientations while checking for clashes [37]. If a clash is found, the last component is rearranged until the cell is free of clashes. Following the initial placement in the box, an additional stage of modification was applied to produce the final placement where the energy of the final configuration of the structure was minimized using the OPLS_2005 force field. This results in a unit cell refined to produce the densest disordered system.

The resulting structure was then prepared for replica exchange molecular dynamics (REMD). Five replicas of the system were simulated in parallel at 290 K, 310 K, 330 K, 350 K and 370 K. Prior to starting the simulations, a materials relaxation protocol was selected, which consisted of a 100 ps NVT Brownian minimization (10 K), a 12 ps NVT minimization (10 K), a 12 ps NPT minimization with restraints on solute heavy atoms (10 K) followed by another 12 ps NPT minimization with restraints on solute heavy atoms and 24 ps NPT minimization with no restraints. After the minimizations, the simulations were conducted for 100 ns with a time step of 2.0 fs at a pressure of 1.01325 bar in an NPT ensemble class. Once the simulations were completed, an out-cms file was generated for each replica. This output file was subsequently opened on Maestro where the plot function was used to generate the RMSD, radius of gyration, solvent-accessible surface area (SASA) graphs and interaction analysis as a function of time.

A probability density function (PDF) was calculated for the radius of gyration (rGyr) and the root mean square deviation (RMSD) values to determine convergence toward the end of the simulation, specifically the last 20 ns of the simulation [38]. PDFs were calculated by counting the number of occurrences at each range of RMSD or rGyr values. The occurrences were then added for each replica and then divided by the total sum. The resulting quotient was then graphed along the ranges of RMSDs or rGyr.

#### 2.1.3. Replica Exchange Molecular Dynamics of Composite Scaffold

Similar to (PAH)_10_, the Disordered System Builder application was utilized to build a randomized multi-component system for the formation of the scaffold. The components of the scaffolds included 6000 water molecules, five polysaccharide peptide conjugates GalC-(LHYQDLLQLQY)_2_, five (FWKT)_4_ peptides, five (PAH)_10_ molecules and five choline acetate molecules in an orthorhombic periodic boundary condition (PBC) that encompassed the entire system with SPC water model [39]. This initial configuration of each component was then selected to be amorphous, which builds the cell by placing each component individually at random locations and orientations while checking for clashes. Similar to (PAH)_10_, if a clash is found, the last component is rearranged until the cell is free of clashes. Following the initial placement in the box, an additional stage of modification was applied to produce the final configuration where the energy was minimized using the OPLS_2005 force field. The final product results in a unit cell refined to produce the densest disordered system.

The resulting structure was then prepared for REMD. Once again, five replicas of the system were simulated in parallel at 290 K, 310 K, 330 K, 350 K and 370 K. Prior to starting the simulations, a materials relaxation protocol was selected using the same conditions that were utilized for the (PAH)_10_ simulation. Once the simulations were completed, we generated the RMSD, radius of gyration, solvent accessible surface area (SASA) data and interaction analysis as a function of time. We also conducted cluster analysis using the Cluster Analysis Calculation panel for the last 20 ns of the simulation.

In addition, radial distribution function (RDF) was also analyzed using Maestro’s Radial Distribution Function panel for each REMD replica for the entire simulation. Specifically, the RDF calculates the probability of finding a particle or interaction at a distance r from each other giving us a “local-view” of the structure [40]. This function is calculated through the trajectory (out-cms file) that is loaded onto the Radial Distribution panel in which the inter-particle distance is calculated for each frame of the trajectory and is then given as a probability. The interactions between the sidechains and functional groups of hydroxyl, carbonyl, carboxyl and amide were analyzed. The SMILES code was used to specify the groups analyzed. To calculate the pair correlation function, a Δr of 0.1 Å was used.

Following the REMD simulations, laboratory analysis was conducted.

### 2.2. Laboratory Studies

#### 2.2.1. Materials

The peptides LHYQDLLQLQY, (FWKT)_4_ and (PAH)_10_ were custom ordered from GenScript (Piscataway, NJ, USA). Primary aortic smooth muscle cells (PCS-100-012), vascular cell basal medium (PCS-100-030), vascular smooth muscle growth kit (PCS-100-042) and 1× Dulbecco’s phosphate-buffered saline (PBS) were purchased from ATCC (Manassas, VA, USA). N-hydroxysuccinimide (NHS), 1-ethyl-3-(3-dimethylaminopropyl) carbodiimide (EDAC), dimethylformamide (DMF), galactaric acid and choline acetate were purchased from Sigma Aldrich (St. Louis, MO, USA). Fetal bovine serum (FBS) was purchased from Neuromics (Edina, MN, USA), and 0.05% Trypsin-EDTA 1×, Quant-iT PicoGreen dsDNA Assay Kit was purchased from Thermo Fisher Scientific (Waltham, MA, USA). WST-8 Cell Proliferation Assay Kit was purchased from Cayman Chemical Company (Ann Arbor, MI, USA). The Caspase-Glo 1 Inflammasome Assay kit was purchased from Promega (Madison, WI, USA) and the Phalloidin CruzFluor 488 Conjugate Antibody was purchased from Santa Cruz Biotechnology (Dallas, TX, USA).

#### 2.2.2. Preparation of Polysaccharide-Peptide Conjugate

Galactaric acid was coupled with LHYQDLLQLQY using traditional peptide coupling methods [41]. Briefly, galactaric acid (1 M) was dissolved in dimethylformamide (DMF), followed by the addition of EDAC (0.5 M) and NHS (0.5 M) to activate its carboxylic groups. This mixture was then allowed to shake at 100 rpm for an hour at 4 °C. Next the peptide, LHYQDLLQLQY (2 M) was added to the conjugate and allowed to be shaken for 24 h at 4 °C. The product obtained was rotary evaporated and then recrystallized using acetone. The chemical structure of this conjugate was confirmed by ^1^H NMR spectroscopy performed using a Bruker 400 MHz NMR. The solvent utilized was DMSO-d6 with 0.3% TMS. The peaks obtained are as follows: δ 0.9 (d, 48H); δ 1.6 (m, 8H); δ 1.8 (t, 16H); δ 2.0 (m, 24H); δ 2.6 (d, 4H); δ 3.2 (d, 8H); δ 3.5 (d, 4H); δ 3.7 (d, 2H); δ 4.4 (s, 2H); δ 4.5 (t, 16H); δ 4.7 (t, 2H); δ 4.8 (m, 6H); δ 5.0 (s, 2H); δ 6.7 (d, 8H); δ 6.9 (d, 8H); δ 7.0 (s, 12H); δ 7.5 (s, 2H); δ 8.2 (s, 22H); δ 8.8 (s, 2H); δ 8.9 (s, 4H); δ12.5 (s, 2H).

#### 2.2.3. Self-Assembly of Polysaccharide-Peptide Conjugate and Conjugation with (FWKT)_4_

The synthesized Gal-(LHYQDLLQLQY)_2_ (1 M) was self-assembled under aqueous conditions for a period of one week at room temperature, washed and centrifuged with deionized water before further analysis. The product was then reconstituted in water and vortexed for five minutes, followed by the addition of EDAC (0.2 M) and NHS (0.2 M). Then (FWKT)_4_ (1.5 M) was added. The mixture was shaken at 4 °C for forty-eight hours, followed by centrifugation for 30 min and washed with deionized water. This process was repeated thrice to remove any unreacted peptides. The supernatant was discarded, and the pellet obtained was then dried for further analysis.

#### 2.2.4. Incorporation of (PAH)_10_ and Choline Acetate by Layer-by-Layer Assembly

To the Gal-(LHYQDLLQLQY)_2_-(FWKT)_4_ conjugate (1.0 M) we incorporated the sequence (PAH)_10,_ (1.0 M). The assemblies were shaken for 48 h and then centrifuged and washed with deionized water thrice. The centrifugate was stored for further analysis. To the (PAH)_10_ composite assemblies with the Gal-(LHYQDLLQLQY)_2_-(FWKT)_4_, choline acetate (0.1 mg) was added and shaken for 24 h to incorporate choline acetate. The resulting scaffold exhibited translucency and possessed a gelatinous texture. At each step, FTIR spectroscopy was conducted to verify the incorporation of each layer. The final biocomposite scaffold was lyophilized before further use.

### 2.3. Characterization

#### 2.3.1. Circular Dichroism (CD) Spectroscopy

Circular Dichroism (CD) spectroscopy was utilized to examine the secondary structure of (PAH)_10_ over time. The sample was allowed to self-assemble in PBS. Readings were then taken at different periods of time to analyze structural changes. Readings were taken in the far-UV region (190 nm–260 nm) using a Jasco J-1500 CD spectrometer. Each sample was run five times and the result obtained was an average of five spectra.

#### 2.3.2. Differential Scanning Calorimetry (DSC)

To examine the thermal properties of (PAH)_10_, and that of the formed scaffold, DSC analysis was carried out using a TA Instruments Q200 DSC (TA Instruments, New Castle, DE, USA). Each sample (2.5 mg) was weighed in aluminum pans and the lid was pressed on the pan using a Tzero sample press kit. Samples were then inserted into the DSC instrument and allowed to run between 0 and 250 °C under nitrogen flow.

#### 2.3.3. Rheology

Rheology measurements of the scaffold were carried out using a Discovery Hybrid HR2 Rheometer (TA instruments, New Castle, DE, USA). To determine elastic and viscous properties, the storage (G’) and loss moduli (G”) were measured between angular frequencies of 0.1 and 100 rad/s. Each reading was performed on a stainless steel peltier plate using an 8 mm peltier cone geometry at strain percentages of 1%, 2% and 5%. To determine elastic modulus, stress versus strain curves were plotted. All measurements were carried out in triplicate in air. To determine the mechanical properties of the bioprinted scaffolds, six-day-old bioprinted scaffolds with cells were utilized and carefully mounted on the instrument, and the modulus and mechanical behavior were determined. The complex viscosities of samples with and without cells were compared at different strain percentages.

#### 2.3.4. Scanning Electron Microscopy (SEM)

To determine the morphology of the bioprinted scaffold, SEM analysis was carried out using a Zeiss EVO MA10 model SEM. Prior to imaging, silicon chips (Ted Pella) (9 mm) were washed with ethanol and irradiated with UV light for 5 min. Once sterilized, the scaffold was then bioprinted directly on the silicon ships, which were coated with poly-L-Lysine inside a Corning Costar 6-well plate. The bioprinted scaffold was then fixed with 2% glutaraldehyde in NaHCA buffer (30 mM HEPES, 100 mM NaCl, 2 mM CaCl_2_) for 1.5 h at room temperature. The fixed bioprinted scaffold was rinsed with PBS and then post-fixed with 1% osmium tetroxide in PBS for one hour at room temperature in the dark. The bioprinted scaffold was then rinsed with distilled water and dehydrated through five-minute steps of washing with 25%, 50%, 75%, 95% and 100% ethanol, dried and then imaged using SEM by directly placing the silicon chip onto the SEM stub using a carbon double stick tape. Imaging was carried out at a range of 7–10 kV at varying magnifications in EP mode. Before bioprinting, scaffolds were also imaged. However, those scaffolds were placed directly onto carbon tapes, air-dried and imaged directly.

#### 2.3.5. Atomic Force Microscope (AFM)

Atomic force microscopy was used to determine the morphology of the self-assembled collagen-like peptide (PAH)_10_ as well as the scaffold containing all the components. Each sample was placed on a mica sheet and allowed to air dry. Samples were then imaged at multiple locations using a Bruker multimode 8 atomic force microscope in scan assist mode using a SCANASYST cantilever, with a frequency of 70 kHz, spring constant of 0.4 N/m and tip radius of 650 nm.

#### 2.3.6. Fourier Transform Infrared (FTIR) Spectroscopy

FTIR spectroscopy was conducted to verify the incorporation of each layer during scaffold formation. Spectra were recorded at a range of 1000–4000 cm^–1^ using a ThermoFisher Scientific Nicolet iS50 FTIR. In general, 20 scans per sample were carried out.

#### 2.3.7. Dynamic Light Scattering (DLS)

To confirm the formation and size distribution of (PAH)_10_ assemblies over time, we conducted Dynamic Light Scattering (DLS) using a Zetasizer Ultra from Malvern Panalytical. Briefly, 15 mg of (PAH)_10_ was diluted in 10 mL of 1× PBS and allowed to self-assemble at room temperature. DLS measurements were carried out over a period of time. Samples were diluted as required. Measurements were taken in triplicate.

#### 2.3.8. Fluorescence Microscopy

Fluorescence microscopy was carried out using an inverted AmScope 1500× phase contrast inverted fluorescence microscope with camera, IN480TC-FL-MF and U2-RFLT 100 digital control box. Samples were viewed at various magnifications and excited at appropriate wavelengths. Images were captured using Amlite software and analyzed.

### 2.4. Cell Studies

#### 2.4.1. D Cell Culture

Primary aortic smooth muscle cells (ATCC PCS-100-012) were grown to confluence in vascular cell basal medium (ATCC PCS-100-030), supplemented with the vascular smooth muscle growth kit components (ATCC PCS-100-042), which provide a low serum environment that is ideal for their propagation. The cells were grown in an incubator at 37 °C under humidified conditions and 5% CO_2_. Media was changed every 2–3 days and cells were split into a new flask upon need.

#### 2.4.2. Cytotoxicity Studies

To determine cell viability, a WST-8 assay was conducted. Studies were conducted in a 96-well plate, by plating the cells at a density of 1 × 10^5^ cells per well. Cells were allowed to spread for three hours in the media (200 µL) followed by the addition of 0.01 mg of the scaffold. For control wells, 5 µL of deionized water was added to untreated cells in media (200 µL) instead of the scaffold. The plates were then incubated at 37 °C under 5% CO_2_. Cell viability studies were conducted at varying periods of time. The WST-8 solution was prepared as per the manufacturer’s instructions and was added to each well followed by gentle mixing. After three hours, the contents of the plate were shaken to ensure homogenous distribution, and the absorbance was read at a wavelength of 450 nm using a Biotek Eon plate reader. The assays were carried out at different time points to examine viability. Studies were carried out in triplicate. Statistical analysis was carried out using Student’s *t*-tests to determine *p* values.

#### 2.4.3. DNA Quantification Assay

To examine whether the scaffold was able to encourage the proliferation of cells, we conducted a DNA Quantification assay by comparing the DNA content of cells grown over different periods of time. The reagent used for this assay was the Quant-iT PicoGreen dsDNA reagent, which specifically detects the fluorescent-stained nucleic acid while minimizing the fluorescence contribution of single-stranded DNA and RNA [42]. Specifically, the assay was carried out at 48 h and 144 h to examine proliferation over time. Cells were grown in Corning Costar 6-well plates at a density of 1 × 10^6^ cells per well with 0.01 mg of scaffold. Control cells were grown without a scaffold. At the designated time point, trypsin was added to each well followed by centrifugation. Next, the pellet of cells was reconstituted in 1 mL of 1 × Tris-EDTA buffer. The contents were then added to a Corning Costar 24-well plate where the cells were lysed using a Fisherbrand^TM^ Model 505 Sonic Dismembrator at 55 Joules to release the DNA. From the plate containing the lysed cells, 100 µL of content was removed from each well and transferred into a 96-well black opaque well plate and 100 µL of Quant-iT Picogreen dsDNA reagent was added to each well. After five minutes at room temperature, the fluorescence of the samples was measured using a Biotek Eon plate reader. Samples were excited at 480 nm. The emission peak at 520 nm was monitored. The results were plotted as a bar graph to quantitatively determine the difference in dsDNA present in each sample. Each study was carried out in triplicate.

#### 2.4.4. Bioprinting

In order to prepare functional three-dimensional scaffolds with precise and controlled microscale structures, we bioprinted the scaffold using a CellInk BioX^TM^ 3D bioprinter. The droplet-based bioprinting method was utilized as it gives better control over the volume of bioink with cells deposited at a predefined location. This method has been successfully adapted for various purposes, including tissue engineering [43]. Prior to bioprinting, we prepared the bioink contents by using a CellMixer kit (Cellink), which contains a cell mixing device that pushes the contents of both syringes (one is designated for the bioink and the other for cells and media) simultaneously into an empty 3 mL cartridge. This device allows for combining an equal amount of scaffold material and cells into the bioprinting cartridge so that there is a homogenous distribution of cells upon bioprinting. Cells were seeded at high density (1 × 10^6^) in flasks and grown to confluence prior to mixing. Once the bioink with cells was prepared, the cartridge was placed in a temperature-controlled print head, followed by the attachment of an 18G 0.5″ stainless steel standard blunt-end needle. The print bed and printhead temperature were then set to 37 °C. Initially, the pressure was set to 5 kPa with an extrusion time of 1 s; however, over time, the pressure was gradually raised to 30 kPa when an increase in extrusion of the bioink was needed. The bioprinter was manually calibrated prior to printing. During the printing process, a circular droplet (5 mm × 5 mm) of the cell-laden bioink was printed onto multiple wells of a Corning Costar 24-well plate. The cell-laden bioprinted scaffolds were then submerged in vascular cell basal medium with growth factors and incubated at 37 °C and 5% CO_2_ until further studies.

#### 2.4.5. Inflammasome Assay

To determine whether the bioprinted scaffold may cause pyroptosis or lead to apoptosis of cells, we performed a Caspase-Glo 1 Inflammasome Assay [44]. The activity of caspase-1 from the cells within the scaffold matrices was measured and compared to that of the untreated 2D cultured cells suspended in a medium. This assay measures caspase activity through luminescence. Active caspase proteins present in each sample cleave the selective Z-WEHD-aminoluciferin substrate. This cleavage then releases luciferin, which reacts with luciferase, causing a luminescence signal. The addition of a proteasome inhibitor, MG-132, allows for a more sensitive detection of caspases as it blocks the proteolytic activity of a proteasome complex that degrades caspase proteins [45]. For this study, however, caspase-1 was of main relevance due to its role as a pro-inflammatory protein that activates cytokines such as interleukin-1β (IL-1β) and IL-8β that are needed to elicit an inflammatory response against pathogens or infection [46]. Therefore, in order to obtain sensitive detection of caspase-1 activity and rule out the activity of other caspase proteins, a caspase-1 specific inhibitor (Ac-YVAD-CHO) was also used [47]. The data were then compared to that of the samples where the inhibitor was omitted in order to determine the specific activity of caspase-1. The bioprinted scaffold containing cells and media were incubated at 37 °C at 5% CO_2_. Two sets of samples were prepared. After seven days, 50 µL of cell culture medium from each sample was transferred to a Falcon white opaque 96-well plate. To the first set of samples, 50 µL of the Caspase-Glo 1 Reagent (containing Z-WEHD substrate in Caspase-Glo1 buffer and MG-132 inhibitor) was added, and to the second set of samples, 50 µL of Caspase-Glo1 YVAD-CHO reagent (containing all of the Caspase-Glo 1 Reagent compounds in addition to the Ac-YVAD-CHO inhibitor) was added. The well plate was then incubated at room temperature for an hour in the dark. The luminescence was then measured using a Biotek Synergy H1 plate reader at an integration time of 1 s, a gain of 240 and a reading height of 6.5 mm.

#### 2.4.6. Cytoskeletal Studies

To examine the effects of the bioprinted scaffolds on the cytoskeleton of the cells, we conducted an actin-cytoskeletal assay [48]. Specifically, we explored whether the bioprinted scaffold conferred a suitable environment for the aortic smooth muscle cells to adhere, proliferate and adapt cytoskeletal cues. Phalloidin CruzFluor conjugate staining solution was used to stain filamentous F-actin. This assay was performed using seven-day-old bioprinted scaffolds. Cells were fixed before imaging. Briefly, the bioprinted scaffolds were incubated with 3.7% paraformaldehyde (methanol-free) for twenty minutes at room temperature, at which point they were washed thrice with 1× PBS. Following fixation, we utilized 0.2% Triton-X100 to permeabilize the cells for ten minutes. Each sample was then washed again with 1× PBS. The staining solution, Phalloidin CruzFluor conjugate was then added and allowed to incubate for 30 min. The cells were then imaged using an inverted phase contrast Amscope IN480TC-20MB13 microscope.

### 2.5. Statistical Analysis

Statistical analysis was carried out using student’s *t*-tests. the *n* value was 3. Data are shown as mean values obtained ± standard deviation.

## 3. Results and Discussion

### 3.1. REMD Simulations for Self-Assembly (Pro-Ala-His)_10_

**Figure 1:**
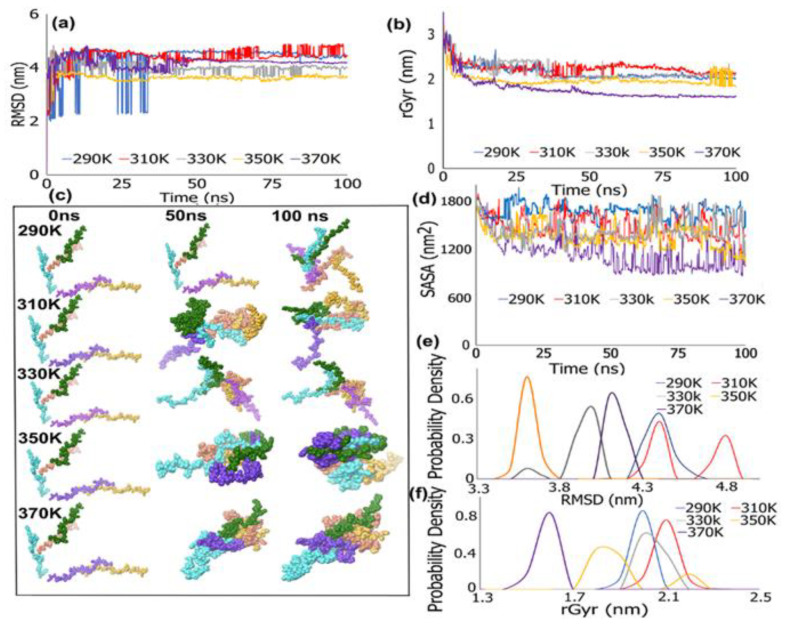
REMD simulations showing the self-assembling ability of (PAH)_10_ at a temperature range of 290 K–370 K over 100 ns simulations: (**a**) comparison of RMSDs; (**b**) comparisons of rGyrs; (**c**) trajectory images at 0 ns, 50 ns and 100 ns. (**d**) Comparison of SASA values; (**e**) Probability distributions of RMSD values of all conformations at varying temperatures for the final 20 ns of the simulation. (**f**) Probability distributions of radius of gyration values at varying temperatures for the final 20 ns of the simulation.

To determine the self-assembling ability of the collagen-like peptide (PAH)_10_, REMD simulations at a temperature range of 290 K–370 K were carried out. This method provides sufficient sampling of the different conformational spaces of peptide-based materials. The root mean square deviation (RMSD) values provide insights into the distance between atoms in proteins, while the radius of gyration (rGyr) indicates the mean-square distance to the center of mass of the system reflecting upon the compactness of the assembly [49,50]. As seen in Figure 1, the RMSD values (Figure 1a) for replica MD at 290 K showed the largest dips ranging from 2.3 nm to 4.4 nm up to 35 ns, indicating changes and disintegrated structures at the beginning of self-assembly while all of the other assemblies were more stable. After 35 ns the peptides reached equilibrium and remained stable throughout the simulation at approximately 4.3 nm. Such initial deviations in RMSD values are expected given the initial nucleation process during self-assembly, which is followed by aggregation resulting in self-assembled structures [51]. These results are corroborated by the rGyr values (Figure 1b), which start at 3.2 nm and gradually decrease to 35 ns. After 40 ns, there is barely any change, and the rGyr remains stable at 2 nm, indicating that the compactness of the assemblies increased over time.

The trajectory images (Figure 1c) show separated structures initially; however, after 50 ns of simulation, the structures are aggregated together with no change in assembly for the rest of the simulation. Unlike the initial deviations seen for the replica at 290 K, the remaining replicas experience relatively fewer atomic deviations. At 310 K initial deviations ranged between 3.6 nm and 4.6 nm for the first 15 ns, followed by smaller subsequent deviations (between 4.3 nm and 4.7 nm) until the end of the simulation. The rGyr shows changes up to 60 ns after which the assemblies appear to become compact. The trajectory image further supports the rGyr data. It is to be noted that at physiological temperature (310 K), the assemblies were found to be integrating into fibrous structures. At 330 K, a similar trend was seen for both the RMSD and rGyr, with small deviations in RMSDs seen up to 80 ns, after which it remains stable at 3.9 nm. This can be confirmed by the trajectory images where no changes in folding are seen between 50 and 100 ns. Therefore, this suggests that these replicas do not undergo significant variations and are therefore stable. For the replica at 350 K, the RMSD shows deviations for the first 10 ns, after which it remains stable at 3.5 nm for the entirety of the simulation. The rGyr, however, showed interesting results, in that the assemblies remained compact, with very little changes, with a steady value of 2.19 nm up to 90 ns, after which it experiences multiple dips in the range of 1.8–2.1 nm, which suggest a change in the folding pattern. This change in folding is seen in the trajectory images where the folding at 50 ns and 100 ns changes from extended to globular. At the highest temperature, 370 K slight deviations in the beginning of the simulation between 3.8 and 4.4 nm are seen. However, after 50 ns the RMSD stabilized at 4.1 nm. The rGyr at 370 K has the least fluctuations and shows the lowest value (1.58 nm) compared to other replicas, indicating that it was the most compact. One would expect that as the temperature increases, further disintegration of the assemblies may occur. However, that was not the case for (PAH)_10_.

The formation of globular, larger structures at 350 K is observed. This may be due to the fact that, at a higher temperature, H-bonding interactions between the (PAH)_10_ and the surrounding water molecules are reduced, resulting in less hydration and change in the folding pattern that causes intramolecular and intermolecular interactions between the (PAH)_10_ molecules to play a greater role, leading to the formation of globular, micelle-like structures [52,53]. Remarkably, at 370 K, large compact fibrillar assemblies are seen, indicative once again of a conformation change. These aggregated structures however are more compact compared to those seen at lower temperatures. A similar pattern was seen for MD simulations of peptide amphiphiles, where in some cases a more stable RMSD was observed at higher temperatures compared to intermediate temperatures [54]. This behavior at 350 K may be attributed to the changes in electrostatic interactions and H-bonding interactions, which may result in a conformation where the thermal energy provided causes changes in folding patterns but may not be adequate to reach a more stable compact structure, which is corroborated by the fluctuations seen toward the end of the simulation in the rGyr at 350 K. The presence of such intermediate structures in assembly formation has also been reported in previous work [55]. Given that the rGyr at 370 K is the lowest and most compact, these results suggest that a transition to higher temperatures may increase the compactness of the assemblies.

To further investigate this behavior of (PAH)_10_ assemblies, the SASA values were analyzed for the entire simulation for each replica. Results are shown in Figure 1d. As can be seen, overall, even though fluctuations are seen throughout the course of the simulation in all cases, we observed that the SASA values reduced over time. Additionally, the SASA values decreased as the temperature was increased; thus, the SASA values at the end of the simulation were lower for the 370 K and 350 K replicas and highest for the 290 K replica, which further corroborates the results discussed above. The values at 310 K and 330 K were in the intermediate range, indicating that the conformations at those temperatures are relatively similar and that, therefore, the solvent-exposed surface areas are also similar. A reduction in SASA values over time indicates that the assemblies are aggregating and becoming less exposed to the surrounding solvent as the simulation progresses. In the case of (PAH)_10_, higher temperatures appear to promote the process of self-assembly due to lesser interactions with water.

We further examined the probability densities to assess the convergence of the local structures formed during the final 20 ns of MD simulations based on the RMSD and rGyr values calculated from the trajectories. As can be seen in Figure 1e, a single narrow peak with the lowest RMSD is seen at 350 K, indicating a highly populated conformation formed in the last 20 ns; smaller peaks with lower probability densities (<0.005) are also seen, indicating negligible subpopulations. Similar results are seen at 370 K, at a slightly higher average RMSD value (4.1 nm). At 290 K, a single peak is observed. However, the peak is much broader, which indicates the appearance of new subpopulations that may be of a different conformation seen to the right side of the distribution. Quantitatively, although this subpopulation is of a significantly lower probability density, the results at 310 K and 330 K show a bimodal graph, indicating two distinct populations, particularly at 310 K. The probability densities are 0.42 and 0.32 for the two peaks seen at 310 K at RMSD values of 4.35 nm and 4.8 nm, indicating two separate conformations are formed to a similar extent, whereas the peaks at 330 K show probability densities of 0.52 and 0.07 at 4.1 nm and at 3.6 nm RMSD values. This indicates that the peak at the RMSD value of 3.6 nm is representative of a much smaller population at 330 K. For the rGyr, (Figure 1f), the probability density peaks observed are single but broad in all cases with the exception of 350 K, where a bimodal peak is observed, which corroborates with results described earlier, given that fluctuations are seen in the rGyr at the last 5 ns of the simulation, signifying rearrangement to a different structure. The trajectories between 80 ns and 100 ns undergo a change to globular conformation at 95 ns. As expected, the population at 370 K shows a more compact structure compared to those seen at lower temperatures. This is likely due to the differences in interactions that occur at the different temperatures. This observation clearly indicates that the convergence adopted by (PAH)_10_ at 370 K is much higher when compared to assemblies seen at other temperatures. By the end of the simulation, this replica has the lowest RMSD, which is corroborated by the probability density of RMSD, which has the leftmost peak, suggesting that during the last 20 ns of the simulation this replica experienced the lowest conformational fluctuations [56].

### 3.2. I-Tasser

We utilized the webserver i-Tasser to predict the secondary structure of (PAH)_10_. The predicted structure was found to be coiled for every residue of the peptide. To arrive at this conclusion, the webserver selected models that closely resembled the inputted template sequence and assigned confidence scores to each one (C-score) ranging from −5 to +2. A high C-score indicates a model with high confidence. The highest C-score given to the top model predicted for (PAH)_10_ by i-Tasser was −1.70. In addition, i-Tasser also assigns a template modeling score (TM), which is usually in the range of 0–1 and provides a structural comparison between the predicted and the template structure. The results obtained for the top model was 0.51 ± 0.15, which indicates that the model had correct topology (TM score > 0.5) as opposed to a TM score of < 0.17, which indicates random similarity [57].

### 3.3. CD Studies

**Figure 2:**
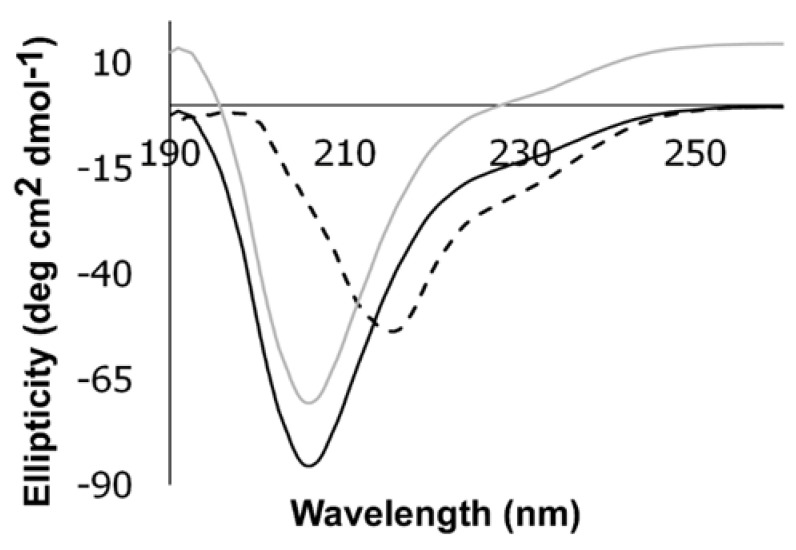
CD spectra of self-assembling (PAH)_10_ over time. ---, 2 days; -,10 days; **—,** 16 days.

To validate the computational results, we performed laboratory studies to examine the self-assembly of (PAH)_10_. In order to determine the secondary structural changes occurring during the self-assembly process of (PAH)_10_, we carried out CD spectroscopy. As shown in Figure 2, the CD spectra indicate that, over time, the peptide formed a coiled structure, which matches the predicted result from I-Tasser. Furthermore, we observed a shift in the negative peak from 216 nm to 206 nm over time, which is typical of poly(pro)II conformation and shows oligomer formation [58]. Although proline in itself has been known to disrupt both alpha-helix and beta-sheet formation, it has also been shown that proline has a propensity to potentially promote helical structures in a hydrophobic environment [59]. In the case of (PAH)_10_, the presence of repeats of His and Pro amino acids separated by the reactively hydrophobic residue Ala promotes self-assembly through strong inter- and intra-molecular H-bond interactions between the -C=O--NH amide groups of the peptide chains (Supporting information Figure S1). Furthermore, the imidazole ring of His can display numerous interactions, including π-π stacking and cation-π interactions. Other work has also demonstrated the π-stacking properties of imidazole, which promotes assembly [60]. Proline, a pentacyclic amino acid with a pyrrolidine ring system on the other hand is conformationally constrained [61] and may induce the formation of polypro coils, which stabilizes the structure [62].

### 3.4. AFM Imaging and Dynamic Light Scattering Analysis

**Figure 3:**
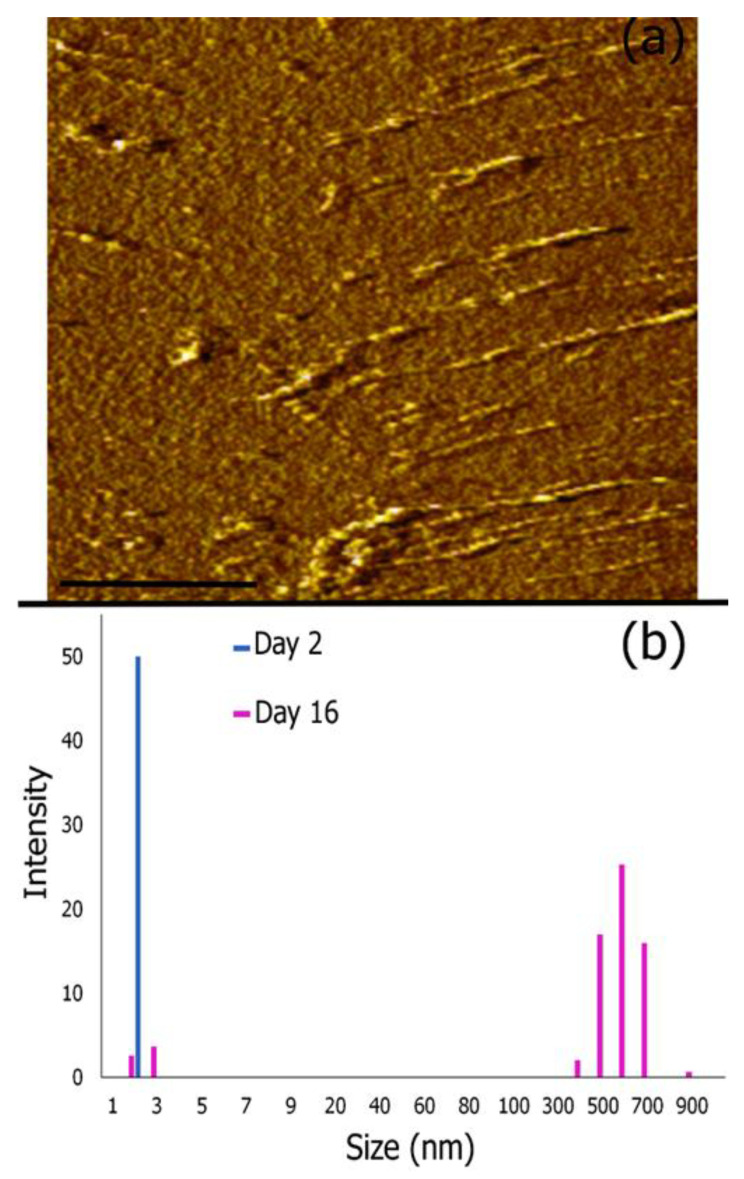
(**a**) Amplitude AFM image of (PAH)10 fibrils grown under aqueous conditions over a period of 2 weeks. Scale bar = 1 µm. (**b**) Dynamic light scattering analysis confirms the growth of assemblies over time.

The formation of assemblies was also confirmed by AFM microscopy and dynamic light scattering studies (Figure 3). As shown in Figure 3a, we observed the formation of fibrillar structures with an average diameter of 100 nm. In some cases, the fibrils appear to aggregate forming supramolecular structures, promoted by the H-bonds as well as stacking interactions. Similar structures were reported for charged pentapeptide collagen mimics containing naphthoxy acetyl-capped diphenylalanine (NapFF)-GDO and (NapFF)-GKO moieties [63]. The authors reported that assembly formation was pH-dependent based on the charge of the residue (K or D). The formation of the (PAH)_10_ assemblies was likely promoted through CH-π interactions between proline and histidine as well as amide–amide H-bond interactions. The growth of assemblies was further confirmed by DLS (Figure 3b).

As seen in Figure 3b, the assemblies after two weeks of growth show a bimodal distribution of nanostructures due to the smaller nanoassemblies representing the monomers and smaller oligomers (size range of 2–4 nm) and the larger nanoassemblies representing nanostructures with an average size of 600 nm. In comparison, the DLS results after one day of assembly showed a unimodal distribution with nanoassemblies in the size range of 2–3 nm. These results confirm the formation of assemblies of (PAH)_10_ over a period of two weeks.

### 3.5. Formation of Multi-Layered Biomimetic Scaffold

#### REMD Simulations of Biomimetic Scaffold

In order to explore the application of the (PAH)_10_ fibers as functional biomaterials, we incorporated (PAH)_10_ with the newly designed amphiphilic peptide conjugates and examined the ability of scaffold formation both computationally and through laboratory analysis. In contrast to conventional molecular dynamics simulations which cannot explore the whole conformational state as it can become trapped in local minimum-energy states, REMD allows for a more error-free representation of the conformational space by overcoming high energy barriers through a combination of MD simulations and Monte Carlo algorithms [64]. This method has gained tremendous popularity in many areas of biomedical simulations including protein folding studies, protein–protein interactions and drug design, among others [65,66]. We chose to simulate a relatively small system due to the significantly long simulation times needed. The components of the scaffold included five molecules of galactaric acid (GalC) conjugated with the peptide LHYQDLLQLQY at both ends, five molecules of the anti-inflammatory peptide (FWKT)_4_, five molecules of (PAH)_10_ and five molecules of the choline acetate ionic liquid molecules, which were put in a box with 600 molecules of water to provide a solvated environment. The temperature range studied was 290 K–370 K. The results of the RMSD values obtained are shown in Table 1; the lowest RMSD value was seen at 330 K, while the values obtained at other temperatures ranged from 6.27 nm to 6.41 nm, implying very little difference and that the most stable structure was obtained at 330 K.

**Figure 4:**
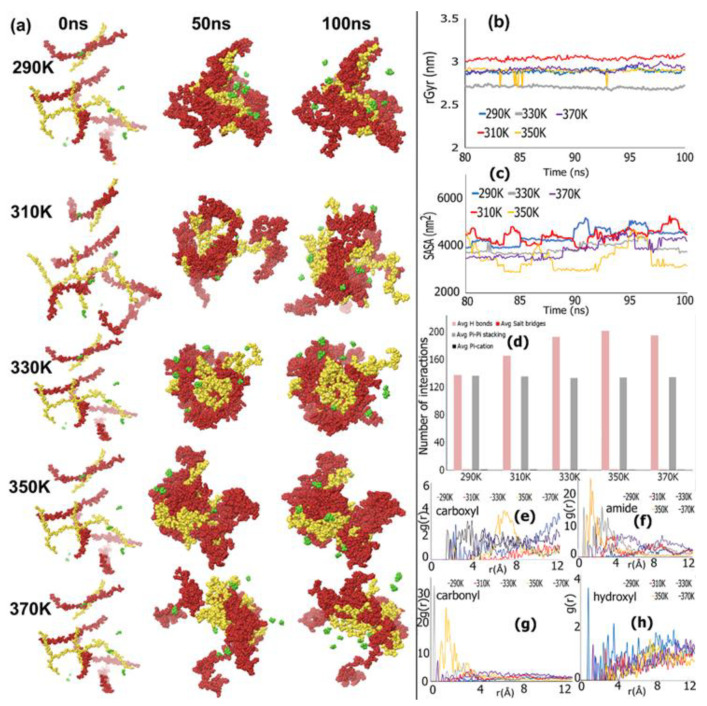
(**a**) Trajectory images of scaffold components at 0 ns, 50 ns and 100 ns at varying temperatures. (Brown represents GalC-peptide conjugates, yellow represents (PAH)_10_ and green represents choline acetate molecules.) (**b**) The radius of gyration represents the scaffold over the last 20 ns of the simulation. (**c**) Comparison of SASA values at varying temperatures in the last 20 ns of the simulation. (**d**) Comparison of the number of various interactions including pi-cation, H-bond and hydrophobic interactions. (**e**) Comparison of radial distribution functions (RDFs) showing (**e**) carboxyl group, (**f**) amide group, (**g**) carbonyl group and (**h**) hydroxyl group interactions.

To further decipher these results, further analysis of the trajectories, SASA values, rGyr, radial distribution functions and types of interactions involved were explored (Figure 4). We first examined the trajectory images at the 0, 50 and 100 ns time points of the simulation. Results are shown in Figure 4a. Across all temperatures, the components of the scaffolds appear to come together and form an aggregate by the end of the simulation; however, the structures seen at 330 K and at 350 K appear to be most compact with the least amount of changes between 50 ns and 100 ns. At 370 K, however, the biocomposite appears to be the least stable and partially disintegrated by the end of the simulation. This is in contrast with what was seen for the (PAH)_10_ fibrils, likely due to the presence of multiple components in the scaffold, which are largely interacting through inter- and intramolecular interactions.

At 290 K, the structures appear to form trapezoid-like shapes with minuscule changes between 50 ns and 100 ns, while an oval/micellar structure with extensions is seen at 310 K. Overall, even though there was no apparent trend between the results at different temperatures, it was determined that all the replicas were able to achieve higher degrees of compactness by the end of the simulation with the exception of 370 K. This is in line with expectations since higher temperatures lead to increased atomistic fluctuations [67]. Figure 4b shows the rGyr of the biocomposite scaffolds for the last 20 ns of the simulation. The lowest rGyr is seen at 330 K, which also corroborates with the trajectories. Overall, the variation in rGyr across the temperatures ranged from 2.7 nm to 3.00 nm, suggesting relatively fewer variations in the compactness of the final assembly formed at the end of the simulation.

We also explored the Solvent Accessible Surface Area (SASA) of the scaffold (Figure 4c) as a function of time for the final 20 ns of the simulation [68]. As can be seen, all of the composites showed fluctuations over time, indicating changes in folding as a result of different degrees of solvent exposure. Lower SASA values were seen at 330 K and at 350 K toward the end of the simulation, indicating a more closely packed structure with less solvent exposure, although the fluctuations seen are higher at 350 K compared to 330 K. The composite at 370 K shows an interesting trend, where the SASA appears to increase with time. This indicates that the assemblies may be unfolding with time. The composites at 290 K and at 310 K show fluctuations throughout the last 20 ns with a slight increase in SASA at the end of the simulation compared to that observed at 80 ns, indicating that those composites may become more solvent-exposed over time due to a more extended structure.

To examine the interactions responsible for the aforementioned results, we explored the hydrogen bonding, and pi-pi stacking, pi-cation and salt bridge interactions that occurred between the components during the entire simulation. Results are shown in Figure 4d. Overall, average π–π interactions remained similar across all temperatures and ranged from 132.9 to 136.1. The highest π–π interaction was seen at 290 K (136.1), while the lowest (132.9) was seen at 330 K. The H-bonds between the components, however, showed interesting changes and were found to increase as the temperature was increased. This implies that H-bonds play a critical role in the formation and stability of the scaffold. As seen in the figure, the highest average number of H-bonds (200.9) is seen at 350 K, while at 370 K there is a slight decrease (194.1). This is likely due to the increased thermal motion, which may promote a larger sampling of interactions. Comparatively, the least number of H-bonds was seen at 290 K (137.2). These results corroborate with the SASA results, where it was shown that, at 290 K and at 310 K, the scaffold formed is more solvent exposed and, therefore, may tend to have more interactions with water rather than with the components of the composite. It is well known that H-bonding is higher in water at lower temperatures and, therefore, given the extended structures formed at those temperatures, the number of H-bonds between the components is relatively less. This can be supported by the trajectory images, which show that, at the end of the simulation, there are multiple chains that are loose and not interacting with the “core” of the scaffold. At the highest temperature studied here, 370 K, a slight decrease in H-bonds is observed, which can be attributed to minor the disintegration of the scaffold at this temperature. The replicas at all temperatures exhibited pi–cation interactions.

We also explored the radial distribution functions (RDFs) [69]. The RDFs of the center of mass between the amide (-NH–-O=C-), carboxyl (-COOH–-HOOC-), carbonyl (C=O---C=O) and hydroxyl (-OH–-OH-) interactions can be seen in Figure 4e–h. Overall, it can be concluded that temperature influences not only the peak heights but also the position of the peaks and, therefore, the interaction distances. Overall, the highest peak heights and relatively narrow peaks are seen for the biocomposite scaffolds formed at 330 K and at 350 K for the carbonyl and amide group interactions, suggesting that those interactions play a significant role in the formation of the scaffold. Specifically, the most well-defined peak was seen between 0.3 Å and 0.5Å for the carbonyl group interactions at 330 K, followed by that at 350 K; however, the peak was broader and shifted to the right 0.8–1.9 Å at a lower intensity. The remaining replicas contain a more diffused distribution of carbonyl group interactions. This pattern of exhibiting stronger interactions at higher temperatures can be justified by the increased thermal motion, which increases the likelihood of finding neighboring atoms, thus leading to interactions [70]. For the amide interactions’ RDF graph, relatively higher peak heights were seen, for the replicas at 330 K, 350 K and 370 K, between 0 and 2 Å, while, at the lower temperatures, shorter peaks were seen along with a more diffused distribution of neighboring amide groups. However, the RDF peaks at 0.8 Å and at 3.5 Å for hydroxyl interactions at 290 K were highest at 290 K suggesting that these group interactions were most prevalent and stronger at the lowest temperature. This defined peak due to hydroxyl group interactions is lost in magnitude and position as the temperature increases, resulting in broader peaks and higher radial distances [71]. The carboxyl group interactions revealed less defined peaks, suggesting that the carboxyl interactions were weak and did not contribute significantly to cluster formation. Specifically, the most pronounced peak occurred for the replica at 350 K; however, its “r” value ranged from 5.4 to 8.5 Å.

**Figure 5:**
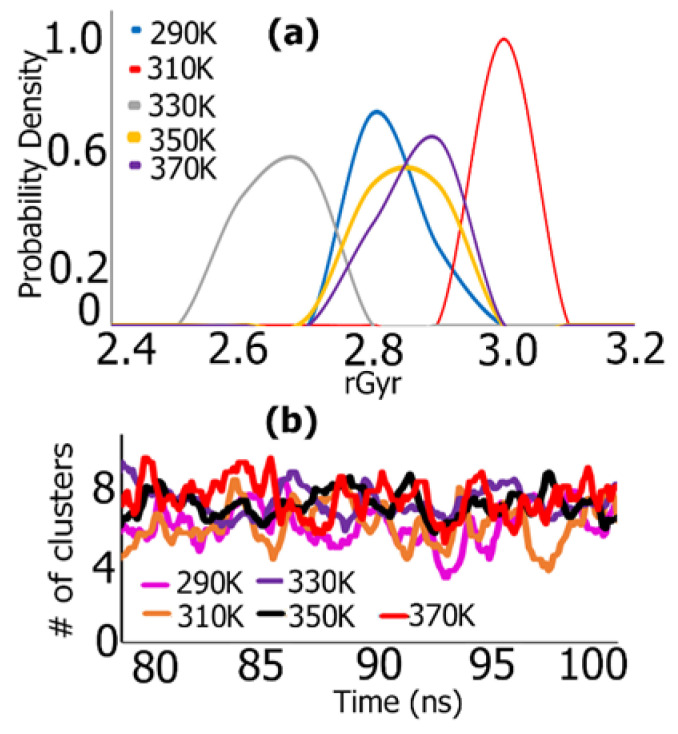
(**a**) Probability distributions of the radius of gyration values of biocomposite scaffold at varying temperatures for the final 20 ns of the simulation. (**b**) Cluster analysis over the final 20 ns.

To further investigate and characterize the existence of the different configurations representative of the scaffold, we have concatenated the Rg values obtained for each replica and determined the probability distributions of Rg values followed by Clustering Analysis (Figure 5). One of the primary observations was that the Rg values of all replicas (Figure 5a) varied within similar ranges (from 2.5 to 3.1 nm). Furthermore, a single broad peak was observed for all the replicas. The scaffold Rg seen at 350 K, 370 K and 290 K appeared in the interval between 2.8 nm and 2.9 nm as displayed by the peaks in that range, indicative of a similar structural population at those temperatures. However, the Rg value seen for the 330 K replica was smaller (2.7 nm) but still broad, suggesting that, at 330 K, a relatively more compact structure is observed; however, there may be small amounts of subpopulations, particularly to the right end of the peak that intersects with the peaks described earlier. Interestingly, the peak seen for 310 K was unique in that it displayed the tallest peak and highest Rg value at 3.0 nm, though still relatively broad. This may be indicative of structural variability, which matches with the trajectories. Overall, because each of the components of the scaffold is non-covalently associated, their relative spatial locations in one configuration may vary from those of another. Similar variations have been reported for REMD simulations conducted for amyloid beta (16—22) octamers [72]. Nevertheless, from a detailed analysis of the replicas, we can conclude that a narrow range of Rg values seen across all the temperatures may correspond to heterogeneous structures, which may contribute to general compactness and varied structural assembly patterns.

Cluster analysis in REMD simulations exhibits the exchange effect of topologically similar structures and categorizes samples so as to improve the homogeneity of structures within a specific category and reduce the heterogeneity between the different categories [73,74]. The clustering analysis results (Figure 5b) for the scaffold over the final 20 ns of the simulation revealed that on average four to ten clusters were seen. At 290 K and at 310 K, the number of clusters was found to be relatively less (four) at 93 ns and at 97 s, respectively. The number of clusters for replicas at all other temperatures ranged from seven to ten clusters. Most importantly, at the end of the simulation, the number of clusters was found to be six at 290 K, five at 310 K, eight at 330 K, nine at 350 K and eight at 370 K. Overall, these results suggest that, at lower temperatures, the components of the scaffold may associate and form lesser number of configurations, which are slightly less varied compared to those at higher temperatures. Overall, higher temperatures resulted in more clusters compared to the clusters at lower temperatures.

### 3.6. Synthesis and Assembly of Biomimetic Scaffold

**Figure 6:**
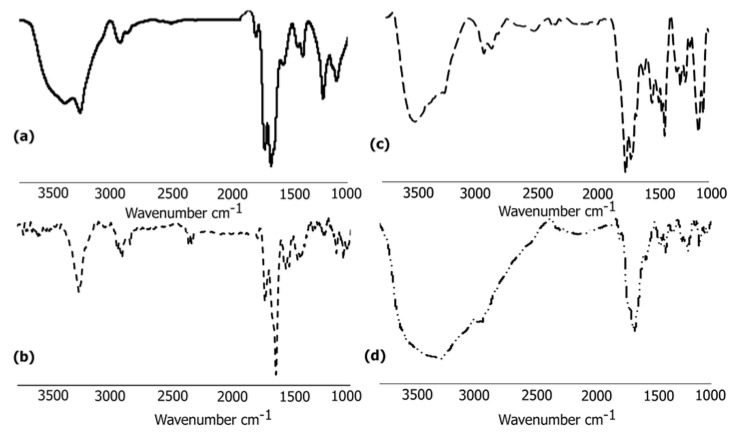
Comparison of FTIR spectra upon incorporation of each layer into the scaffold. (**a**) GalC-(LHYQDLLQLQY)_2_ assemblies; (**b**) GalC-(LHYQDLLQLQY)_2_ assemblies conjugated with (FWKT)_4_; (**c**) (PAH)_10_ incorporated with GalC-(LHYQDLLQLQY)_2_–(FWKT)_4_ assemblies; (**d**) choline acetate incorporated with GalC-(LHYQDLLQLQY)_2_–(FWKT)_4_,-(PAH)_10_ assemblies.

In order to validate if the biocomposite scaffold displayed cytocompatibility and could potentially be developed as a biomaterial, we synthesized the scaffold by conjugating the LHYQDLLQLQY peptide with galactaric acid (GalC), self-assembled it and further conjugated the (GalC- LHYQDLLQLQY)_2_ assemblies with the anti-inflammatory peptide (FWKT)_4_. It was conjugated to the assemblies by coupling the free carboxylic groups of the peptides at both ends with the free amino groups of (FWKT)_4_. The components were again allowed to self-assemble and then a layer-by-layer assembly approach was utilized to incorporate the (PAH)_10_ self-assembled nanofibers. GalC was efficiently functionalized with the LHYQDLLQLQY peptide at both ends through amide coupling, which resulted in the formation of amphiphilic assemblies, due to the hydrophilic -OH groups present on the GalC moiety and the peptide sequences containing multiple hydrophobic amino acids such as tyrosine and leucine at the two ends. The self-assembly was promoted through hydrophobic interactions, stacking interactions as well as H-bonding interactions as shown in the computational studies. Furthermore, interactions between the amide groups of the peptides (both intra- and intermolecular) promoted self-assembly. Finally, the biocompatible ionic liquid choline acetate was incorporated into the gel. The formation of the assemblies was investigated through FTIR spectroscopy (Figure 6).

#### 3.6.1. FTIR Analysis

The formation of the scaffold was confirmed through FTIR spectroscopy. The GalC-(LHYQDLLQLQY)_2_ amphiphilic assemblies displayed strong amide I peaks at 1695 cm^−1^, 1650 cm^−1^ and at 1630 cm^−1^. The amide II peak was seen at 1550 cm^−1^, while the C-O stretching peak was found to be at 1220 cm^−1^. The appearance of the peak at 1630 cm^−1^ can be attributed to the presence of the tertiary amide groups due to glutamine residues within the peptide, while the peak at 1650 cm^−1^ is due to the secondary amide groups that make up the peptide backbone as well as the amide bonds formed upon conjugation to the carboxylic acid groups of galactaric acid. The peak at 1695 cm^−1^ may be due to turns during assembly formation. In addition, we also observed a strong -OH peak at 3420 cm^−1^ due to the hydroxyl groups of galacatarate and the tyrosine moieties as well as the carboxyl group -OH peaks. The -NH stretching peak is seen at 3277 cm^−1^. The -CH stretching peaks were seen at 2930 cm^−1^ and at 2870 cm^−1^. As expected, the amide peaks were not seen for neat GalC, which showed a strong band at 1735 cm^−1^, characteristic of free carboxyl groups and peaks at 1195 cm^−1^ and at 1400 cm^−1^ attributed to C-O stretching and -OH bending, respectively. Upon functionalizing the GalC-(LHYQDLLQLQY)_2_ amphiphilic assemblies with (FWKT)_4_, we observed further shifts. The amide I bands were seen at 1626 cm^−1^ and at 1719 cm^−1^ while the amide II peak was seen at 1541 cm^−1^ with shoulders at 1557 cm^−1^ and at 1514 cm^−1^. The C-O peak was shifted to 1217 cm^−1^, while the -NH stretch peak was shifted to 3281 cm^−1^. Such shifts are indicative of interactions between the self-assembled GalC-(LHYQDLLQLQY)_2_ conjugates and the (FWKT)_4_ peptide. The appearance of a peak at 1719 cm^−1^ is likely due to the presence of the carboxyl groups of threonine. Similar shifts were seen when poly-L-lysine was incorporated into PNIPAM gels [75]. Additionally, the -OH peak was significantly diminished, indicating their involvement in H-bond interactions. These results confirm the attachment of the (FWKT)_4_ peptide with the GalC-(LHYQDLLQLQY)_2_ assemblies.

We then further functionalized the assemblies with self-assembled (PAH)_10_ by a layer-by-layer assembly approach. This led to the appearance of multiple peaks at 1722 cm^−1^; 1675 cm^−1^ with a shoulder at 1631 cm^−1^ in the amide I region. The amide II peaks were seen at 1566 cm^−1^ and at 1494 cm^−1^. The presence of such multiple broad overlapping bands in the carbonyl region is indicative of the co-occurrence of various supramolecular species [62] confirming the incorporation of (PAH)_4_. Furthermore, of particular note is the strong, broad peak seen in the 3000–3200 cm^−1^ region, with a peak at 3269 cm^−1^ and 3518 cm^−1^ [76]. The peak at 3269 cm^−1^ is due to NH-stretching interactions between the imidazole ring system of His and the indole ring system of tryptophan, indicating the role of those interactions in the formation of the composite assemblies. In comparison, neat (PAH)_4_, showed strong peaks in the carbonyl region at 1713 cm^−1^, 1667 cm^−1^ and 1630 cm^−1^ along the amide II peak at 1537 cm^−1^. Furthermore, peaks were also seen at 1457 cm^−1^ due to -OH bending and at 1389 cm^−1^ and 1213 cm^−1^ due to C-O stretching. The C-H stretching peak was seen at 2930 cm^−1^ (data not shown). The final scaffold after incorporation of choline acetate showed characteristic peaks at 1651 cm^−1^ with a shoulder at 1704 cm^−1^ and at 1556 cm^−1^ in the carbonyl region, while a strong, broad peak is once again seen in the region between 3000 cm^−1^ and 3500 cm^−1^ due to strong H-bond interactions with the scaffold and hydration of the ionic liquid. In previous studies, it has been shown that choline acetate is capable of forming strong H-bonds [77], which is also corroborated by our results. It is likely that choline acetate forms strong H-bonds with the NH- and -C=O groups of the peptide moieties as well as with the -OH groups of the galatarate and Thr components of the scaffolds and electrostatic interactions with the His moieties of (PAH)_10_.

#### 3.6.2. Morphological Analysis of Biocomposite Scaffold

**Figure 7:**
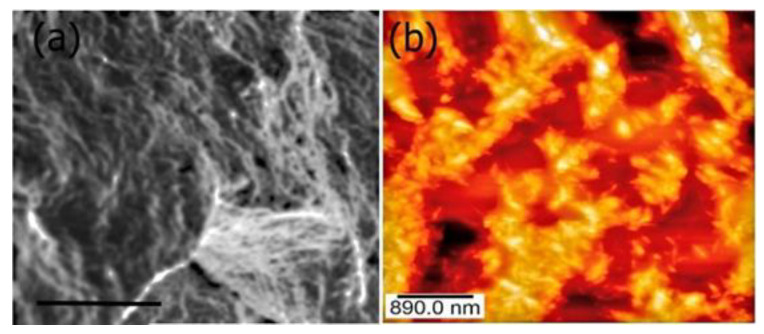
(**a**) SEM analysis of the final biocomposite scaffold formed by a layer-by-layer assembly. Scale Bar = 2 µm. (**b**) AFM Height image showing the multilayered porous structure of the scaffold.

To investigate the morphology of the final scaffold, we carried out both SEM and AFM analysis (Figure 7). As can be seen in the SEM (Figure 7a), a multilayered gelatinous scaffold is formed, which is also corroborated by AFM analysis (Figure 7b), which reveals a fibrous scaffold with several layers and a porous structure. These results confirm that the (PAH)_10_ based hybrid scaffold formed dense fibrillary gelatinous matrices. Such multi-layered scaffolds are desirable for potential tissue engineering applications due to their ability to entrap cells and provide a suitable surface for spreading, growth and proliferation of cells and for the flow of nutrients and blood.

#### 3.6.3. Thermal Analysis of Biocomposite Scaffold

**Figure 8:**
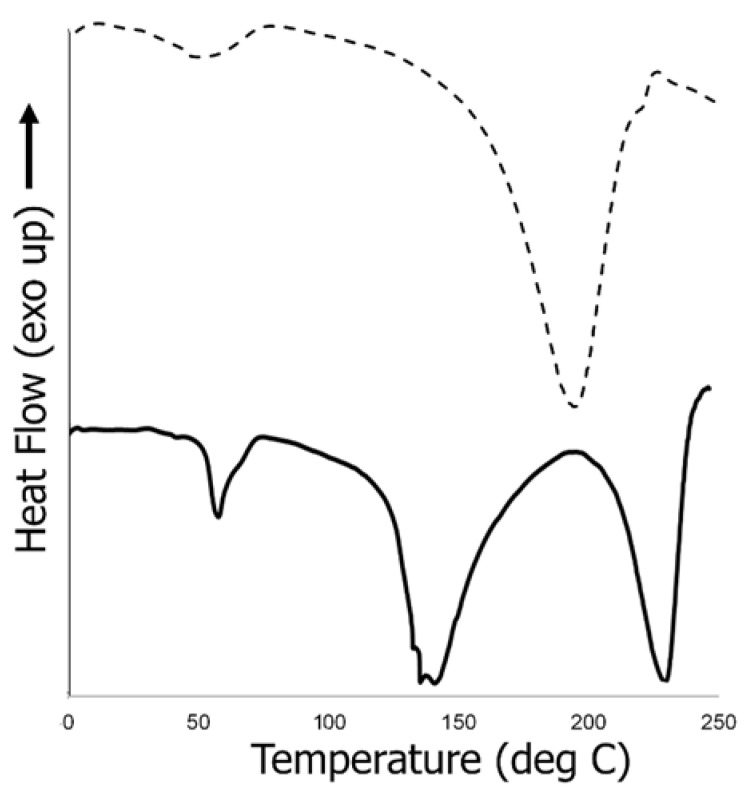
Comparison of DSC thermograms. The dotted line is indicative of the biocomposite scaffold and solid line shows the (PAH)_10_ assemblies.

In order to investigate the thermal properties of the scaffold, we carried out DSC analysis. Results are shown in Figure 8. The most prominent peak observed is an endothermic peak at 196.2 °C, which is attributed to the thermal melting of the scaffold. Additionally, a short endothermic peak is seen at 51.3 °C, most likely due to the unfolding of the (PAH)_10_ assemblies within the biocomposite due to changes in intra- and intermolecular interactions within the scaffold as well as loss of loosely bound water. In comparison, the (PAH)_10_ assemblies alone showed that unfolding occurred at 58.1 °C. The slight difference in temperature observed is likely because of the changes in H-bonding interactions that occur when incorporated into the biocomposite. This peak is akin to the peak observed in the case of native collagen II (seen between 58 °C and 65 °C), where the endothermic peak has been attributed to thermal changes that occur within collagen fibrils due to changes in its triple-helix conformation when heated, due to fluctuations in intra- and inter-molecular hydrogen bonds in collagen [78]. The second broad endothermic peak for (PAH)_10_ seen at 143.6 °C is attributed to the evaporation of tightly bound water, while the thermal melting peak due to degradation is seen at 232.1 °C. In comparison, in the case of native Type II collagen fibrils, the loss of strongly bound water occurs around 150 °C. These results indicate that (PAH)_10_ assemblies are integrated into the scaffold and that (PAH)_10_ assemblies may closely mimic collagen Type II.

#### 3.6.4. Mechanical Properties

**Figure 9:**
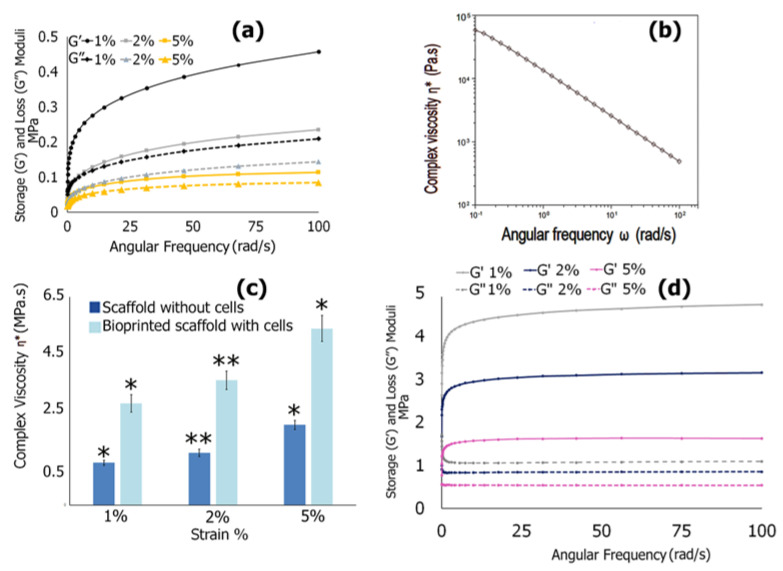
Rheological analysis of biocomposite scaffold with and without cells. (**a**) Comparison of storage and loss moduli of biocomposite scaffolds at 1%, 2% and 5% strain without cells; (**b**) complex viscosity versus angular frequency at 1% strain for biocomposite scaffold without cells; (**c**) comparison of complex viscosity of cell-laden bioprinted scaffolds at different strain percentages; (* *p* < 0.05, ** *p* < 0.01), (**d**) storage and loss moduli of bioprinted scaffolds with cells. *n* = 3 for all cases.

To ensure that the scaffold has suitable mechanical properties, we carried out rheology studies (Figure 9). The response of storage and loss moduli for scaffolds without cells against angular frequency is presented in Figure 9a. The samples were subjected to an angular frequency sweep at varying % strain. Results show that the moduli were dependent upon the % strain applied. The highest storage and loss moduli were seen at the lowest strain (1% strain), and the moduli decreased with increasing strain. This reduction in moduli, when the strain was increased, was attributed to the fact that the gelatinous biocomposite formed three-dimensional aggregate networks, which are affected by the dynamic viscoelasticity of materials that are dependent upon inter- and intra-molecular interactions [79]. At higher strain, it is likely that the distance between the aggregates is increased, resulting in a relatively lower storage modulus, while at lower strain the aggregates are more tightly attached together due to stronger interactions. Overall, the storage modulus increased with frequency in all cases; however, at higher strain (5%), the storage modulus leveled off and remained in the order of magnitude of 1 × 10^5^ Pa. The fact that, at higher frequencies, the storage modulus is higher implies that the biocomposite may become stiffer under those conditions. Additionally, the difference between the storage and loss modulus was significantly higher at a % lower strain than that seen at a higher % strain. Overall, in all cases, the storage modulus was found to be higher than the loss modulus indicating that the biocomposite demonstrates elastic behavior [80].

To determine the shear thinning behavior, we examined the complex viscosity versus angular frequency. Figure 9b shows the shear thinning behavior of the scaffold as shown by the decrease in complex viscosity as the angular frequency was increased at 2% strain. Similar results were obtained at other strain percentages (data not shown). Such shear-thinning behavior is desirable for extrusion-based 3D bioprinting [81]. To examine the differences that occur when laden with cells, the complex viscosity of the bioprinted scaffold with cells was measured and compared with the scaffold without cells as seen in Figure 9c. In general, the number of cells was kept constant at 1 × 10^5^ cells for all samples. As can be seen for the scaffolds with cells, the complex viscosity was found to increase. Additionally, the complex viscosity was found to increase with increasing % strain. This increase observed for the bioprinted scaffold with cells can be attributed to the higher volume fraction in the presence of cells [82]. The elastic modulus was calculated for both acellular scaffold and that of the bioprinted scaffold with cells. The values obtained were 0.19 ± 2.5 MPa and 0.92 ± 3.7 MPa, respectively. A comparison of the storage and loss moduli of the bioprinted scaffold with cells (Figure 9d) at the three different strains (1%, 2% and 5%) demonstrated the same trend as that of the acellular scaffold. Both the storage and loss moduli decreased with increasing strain. Furthermore, an increase in the values of both modulus values was observed, likely due to the increase in elasticity imparted by cells.

### 3.7. Cell Adhesion, Proliferation and Formation of Cell Scaffold Matrices

**Figure 10:**
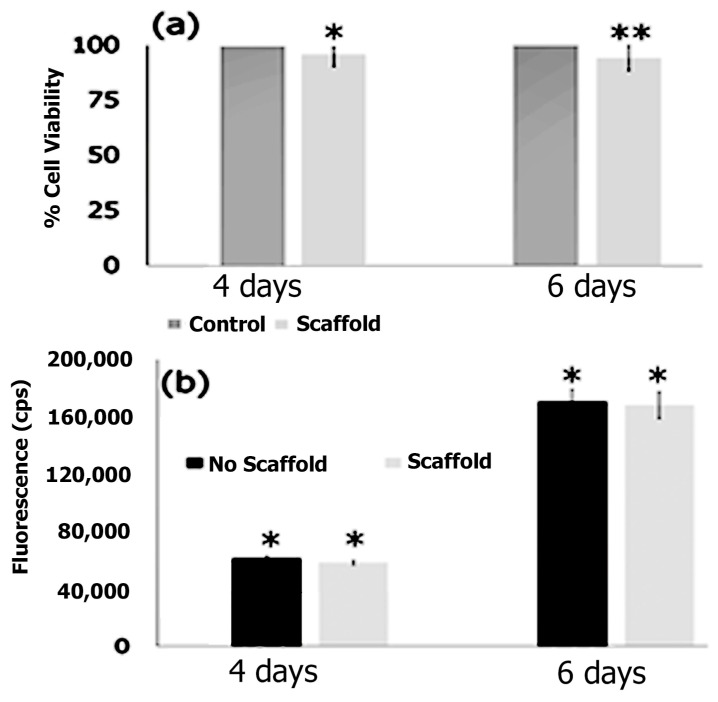
(**a**) WST-8 Assay showing percent cell viability of the aortic smooth muscle cells s at 96 h and at 144 h. (**b**) DNA Picogreen Assay shows an increase in DNA correlated with the fluorescence intensity over time in the presence and absence of the biocomposite scaffold. * *p* < 0.05; ** *p* < 0.01. (*n* = 3).

To ensure cytocompatibility and cell growth over time, we examined if the scaffold displayed cytotoxicity in the presence of mammalian aortic smooth muscle cells. We also explored the growth of cells using a Quant-iT PicoGreen dsDNA Assay, which quantifies double-stranded DNA (Figure 10). We chose this specific cell line as these smooth muscle cells (SMCs) are vascular and are the principal cell type within the aortic wall and play an important role in extracellular matrix regeneration, degradation, proliferation and contractility [83]. Therefore, these cells can provide a model for cellular interactions with the scaffold. The results of the WST-8 cytotoxicity assay are shown in Figure 10a. The growth of cells in the presence and absence of a scaffold (control) at two different time points (4 days and 6 days) is shown. As can be seen, the cells displayed 95% viability after 4 days of growth and 94% viability after 6 days. Thus, the cells continued to proliferate over time, in the presence of the scaffold, which indicated that the scaffold did not display cytotoxicity and is cytocompatible.

To further confirm that the scaffold promoted the proliferation of cells over time, a Quant-iT PicoGreen dsDNA Assay was conducted as it is a reliable strategy for quantifying cell proliferation in 3D systems. [84] As seen in Figure 10b, there is a significant increase in the fluorescence signal of double-stranded DNA over a span of 6 days (144 h), indicating that the scaffold promotes cell proliferation over time. The control sample was compared to the seeded scaffold, which revealed slightly higher proliferation for both time points. While slightly less dsDNA was detected in the presence of the scaffold, it can be justified due to the additional physical barriers presented by the scaffold. The fact that proliferation significantly increased after 6 days, however, suggests that the environment of the scaffold successfully promotes cell proliferation over time.

**Figure 11:**
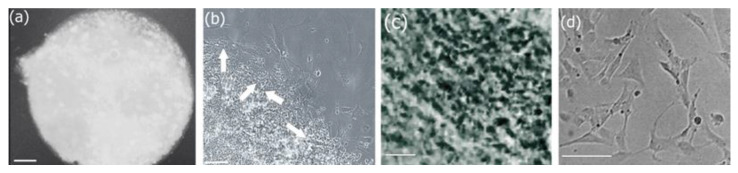
Optical microscopy image of bioprinted scaffold with aortic smooth muscle cells (**a**) immediately after bioprinting (10× magnification) and (**b**) after 48 h of growth (Scale bar = 20× magnification). (**c**) SEM image of bioprinted scaffold showing cells completely embedded within the matrix after 10 days of growth. Scale bar = 5 µm. (**d**) Optical microscopy image of control cells (growth without scaffold) after 48 h. Scale bar = 20 µm.

After confirming cytocompatibility, we then bioprinted the cells with the scaffold using droplet methodology and examined the interactions with the cells. The results of the bioprinted scaffold with cells are shown in Figure 11. The optical microscopy image of the bioprinted scaffold immediately after bioprinting is shown in Figure 11a. As can be seen, a uniform spherical scaffold was observed. After 48 h (Figure 11b), cells were observed both within and on the edges of the surface of the scaffold (as shown by the arrows) where large actin extensions were seen signifying cellular movement and adhesion [85]. They were found to be well spread out, exhibiting fibroblast-like morphology as is expected for aortic SMCs [86]. Additionally, the cells were well distributed within the scaffold matrix. Over time, within 10 days, the cells showed hierarchical organization with cell-to-cell interactions and distribution within the matrix (Figure 11c). These results further confirm that the bioprinted scaffold provides a conducive environment for the cells to proliferate and make critical cell–cell interactions that are necessary for tissue-engineered scaffolds. We also compared the morphology of the control cells (cells grown without scaffold, Figure 11d), which show extended morphologies typical of aortic smooth muscle cells.

**Figure 12:**
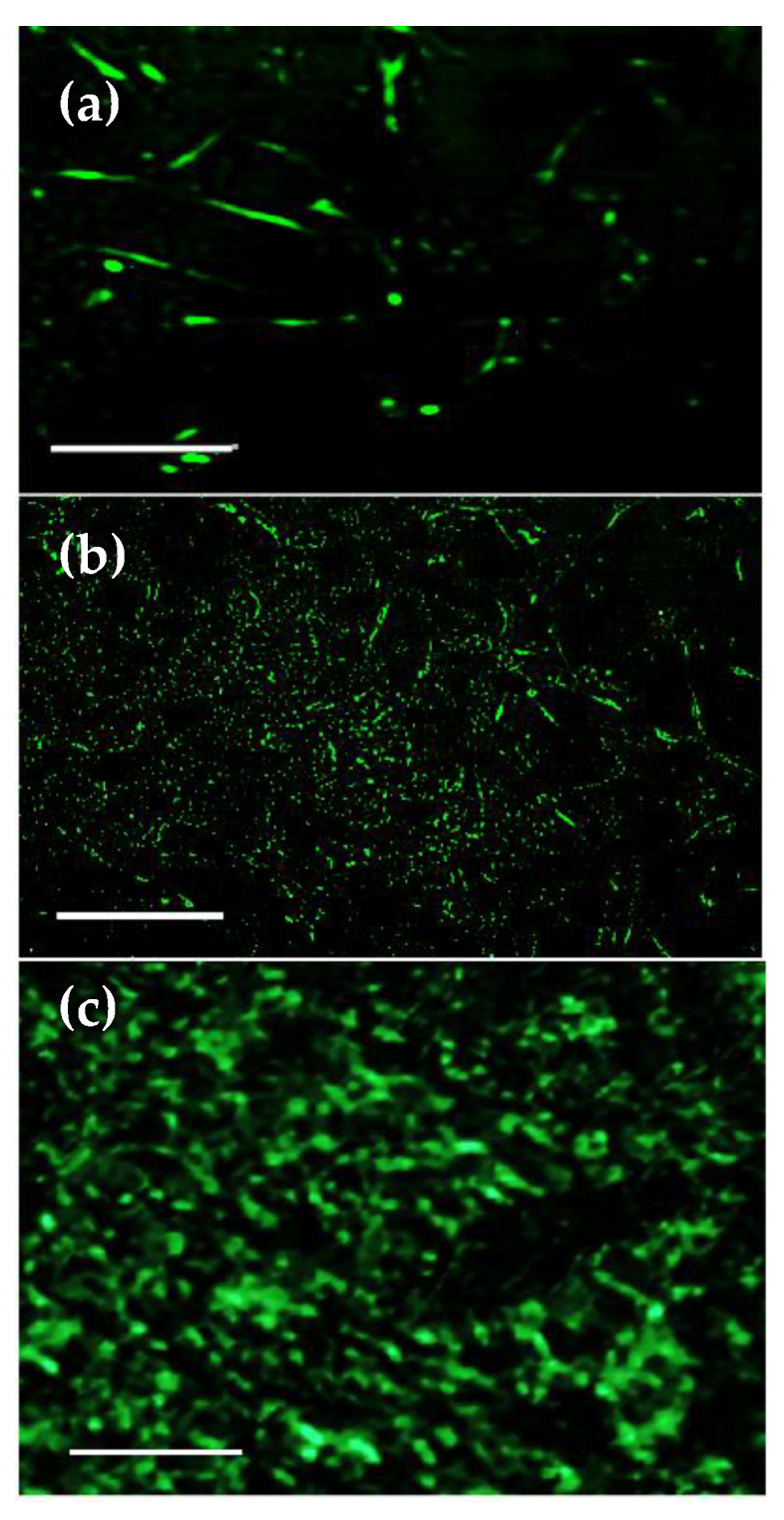
Fluorescence images of cells showing actin cytoskeleton (**a**) on the edges of the bioprinted scaffold after 48 h, (**b**) within the bioprinted scaffold after 96 h and (**c**) within the bioprinted scaffold after 10 days. Scale bar = 50 µm.

To further examine the effects of cytoskeletal dynamics and organization of the aortic SMCs in the bioprinted scaffold, we conducted an actin staining assay using FITC-phalloidin. Cell spreading and formation of actin filaments were confirmed using fluorescence microscopy (Figure 12). As shown in Figure 12a, the images indicate that the adhered cells are elongated and demonstrate lamellar morphology, with cell spreading and extended actin filaments at the edges of the bioprinted scaffold after 48 h. However, cells entrapped within the scaffolds over a period of time are morphologically relatively granular though they also were found to be well-spread and form cell–cell interactions (Figure 12b,c). Thus, the bioprinted scaffold was found to not only promote cell adhesion and proliferation but also provide the appropriate conditions for cell migration that would be essential for tissue engineering.

**Figure 13:**
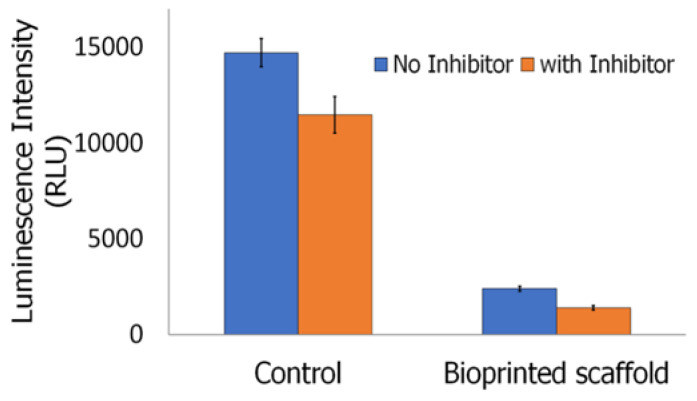
Inflammasome assay showing the luminescence intensity triggered by Caspase I activity. Comparison between control cells and cells in the bioprinted scaffolds.

One of the hallmarks of the early stages of apoptosis is the activation of intracellular proteases such as caspase enzymes, which are involved in the cleavage of protein substrates followed by the disintegration of cell membranes [87]. Caspase-I, in particular, is also called inflammatory caspase, which is activated through inflammasome assembly and is involved in inflammatory response against infections or cellular damages, which in turn activate interleukin (IL)-1β and IL-18 [88]. In order to ensure that the biocomposite scaffold does not elicit an inflammatory response in future applications, we conducted an inflammasome assay. As seen from Figure 13, the luminescence signal for the control samples is significantly higher than that of the seeded scaffold (14,708 vs. 2390 RLU, respectively), revealing that the caspases are more active in the control cells. This difference in caspase activity suggests that the cells within the bioprinted scaffold may have reduced responses to stimuli such as the presence of pathogens, mitochondrial dysfunction, DNA damage, ER stress or inflammatory signaling that indirectly activate the inactive zymogenic precursors of caspases [89].

Although it is not possible to determine the specific caspase that is responsible for the detected caspase activity, we can assess the contribution of caspase-I stimuli [90,91]. Since the samples treated with the Ac-YVAD-CHO inhibitor (in orange) inhibit caspase-I activity by 99% without inhibiting any of the cross-reacting caspases, the luminescence for those samples was subtracted from the luminescence of the samples without the inhibitor. This yields a luminescence of 3247 RLU for the control sample and 994 RPU for the bioprinted scaffold. As seen, the caspase-1 activity for the bioprinted scaffold is significantly lower than that of the control wells, suggesting that the scaffold may potentially mitigate inflammasome formation.

## 4. Conclusions

In this work, we have developed a new bioprinted scaffold for potential applications in tissue regeneration. The scaffold was created by conjugating a pectin derivative, galataric acid with a pro-angiogenic peptide and a cortistatin-derived peptide. The formed assemblies were further functionalized with a novel collagen-like peptide (PAH)_10_ through a layer-by-layer assembly, followed by the incorporation of the bio-ionic liquid choline acetate to form a gelatinous scaffold. The assembly of (PAH)_10_ and its interactions with the galatarate-peptide conjugates were examined both computationally and through laboratory experiments. REMD simulations revealed that the self-assembly of (PAH)_10_ was able to form stable collagen-like assemblies, particularly at higher temperatures. The incorporation of (PAH)_10_ into the scaffolds resulted in stable structures. The replicas were able to attain compactness by the end of the simulation; however, the replica at the highest temperature utilized in the modeling study (370 K) experienced slight disintegration. Experimentally, it was revealed that (PAH)_10_ formed random coiled structures and that it was readily incorporated with the galatarate-peptide conjugates and choline acetate and formed a gelatinous matrix. The formed gel was then bioprinted along with aortic smooth muscle cells to prepare bio-printed cell scaffold matrices. The scaffold was found to be biocompatible, and the cells remained adhered within different layers and continued to proliferate. Furthermore, the formation of actin filaments and elongated morphology was observed indicative of cell migration within the scaffold. Overall, these studies reveal that the collagen-like peptide (PAH)_10_ may have potential applications in tissue engineering applications. Furthermore, this proof-of-concept bioprinted model using galactarate-conjugated to functional peptides along with (PAH)_10_ and a bio-ionic liquid can impart interesting functional properties that may be potentially utilized for further studies for therapeutic applications such as those focused on cell delivery and retention and in future in vivo studies for tissue engineering.

## 5. Future Goals

While the bioprinted scaffold containing (PAH)_10_ and other bio-organic components showed that it could be potentially utilized for future therapeutic applications, there are several future directions that one could pursue depending upon tissue specificity. One may potentially develop these bioprinted scaffolds for developing cardiac patches. For such an application, to mimic the tissue environment, a multi-cellular component can be utilized in, for example, co-cultures of fibroblasts, cardiomyocytes, endothelial and vascular smooth muscle cells. In vitro biodegradation studies can provide further insights into whether the scaffolds can degrade over time, which in turn can offer information about tissue remodeling capability. To further enhance growth, the scaffolds could be subjected to electrical stimulation. Alternatively, mesenchymal stem cells may be utilized, and local production of cytokines and growth factors will provide additional cues as to how the bioprinted scaffold may behave in vivo. Finally, because these are primarily peptide-based scaffolds, one could scale up and bioprint larger scaffolds with varying pore sizes and carry out in vivo studies in mice. Those studies could provide further understanding of the reparative mechanisms triggered by the bioprinted scaffolds.

## Figures and Tables

**Table 1:** Comparison of RMSD values obtained from REMD simulations of the biocomposite scaffold at varying temperatures.

Temperature (K)	Average RMSD (nm)
290	6.28
310	6.59
330	5.71
350	6.29
370	6.41

## Data Availability

The relevant experimental data are within this paper.

## Supplementary Materials

The following supporting information can be downloaded at: https://www.mdpi.com/article/10.3390/biomimetics8070548/s1, Figure S1: Three-dimensional chemical model showing the interactions involved in the self-assembly of (PAH)_10_.
