# Supplementary material for: Development of Self-Assembled Biomimetic Nanoscale Collagen-like Peptide-Based Scaffolds for Tissue Engineering: An In Silico and Laboratory Study
Source: Biomimetics (Basel). 2023 Nov 14;8(7):548. doi: 10.3390/biomimetics8070548 (PMC10669358; doi:10.3390/biomimetics8070548)
Supplement: Supplementary file 1 [file biomimetics-08-00548-s001.zip › biomimetics-2653533-supplementary.pdf]

Supporting Information

Exploring the Development of Hybrid Collagen Like Peptide Based Scaffolds -A Replica Exchange Molecular Dynamics and Laboratory Study

Department of Chemistry, Fordham University, 441 East Fordham Road, Bronx, New York 10458, USA

Beatriz G. Goncalves, Ryan M. Heise and Ipsita A. Banerjee*

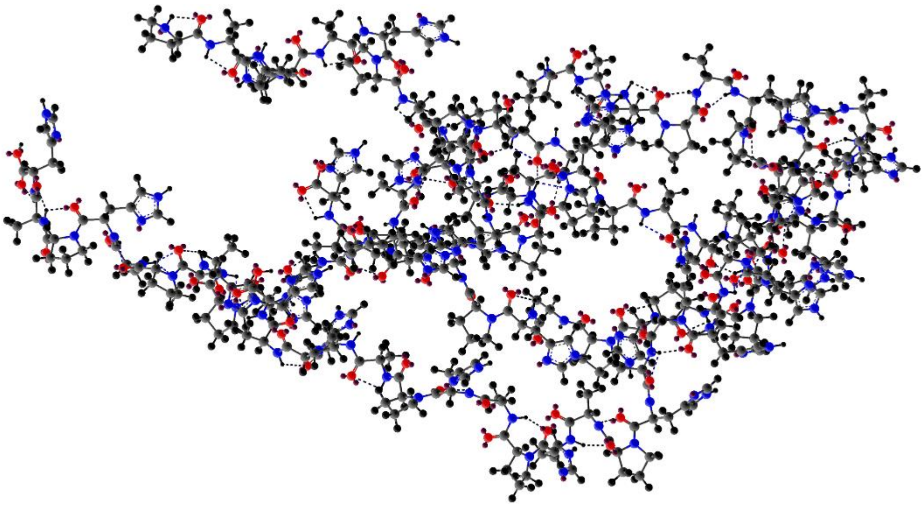

Figure S1. Three dimensional chemical model showing the interactions involved in the self-assembly of $(PAH)_{10}$.
